# Supplementary material for: Tobacco Smoke Exposure in Children and Adolescents: Prevalence, Risk Factors and Co-Morbid Neuropsychiatric Conditions in a US Nationwide Study
Source: Healthcare (Basel). 2024 Oct 22;12(21):2102. doi: 10.3390/healthcare12212102 (PMC11545687; doi:10.3390/healthcare12212102)
Supplement: Supplementary file 1 [file healthcare-12-02102-s001.zip › healthcare-3097932-supplementary.pdf]

Supplemental materials:

Table S1. The National Survey of Children's Health (NSCH) 2020-2021 related Questions list.

Item	Description	Numerator/Response Categories
Prevalence of ADD/ADHD, age 3-17 years (ADHDind_21)	Does this child currently have Attention Deficit Disorder (ADD) or AttentionDeficit/Hyperactivity Disorder (ADHD), age 3-17 years?	Does not have condition; Ever told, but does not currently have condition; Currently has condition
Severity of ADD/ADHD, age 3-17 years (ADHDSevInd_21)	Would you describe this child's current Attention Deficit Disorder (ADD) or Attention-Deficit/Hyperactivity Disorder (ADHD) as mild, moderate or severe, age 3-17 years?	Does not currently have ADD/ADHD; Current ADD/ADHD, rated mild; Current ADD/ADHD, rated moderate or severe
Prevalence of autism/ASD, age 3-17 years (AutismInd_21)	Does this child currently have autism or autism spectrum disorder (ASD) including Asperger's disorder, pervasive developmental disorder?	Does not have condition; Ever told, but does not currently have condition; Currently has condition
Severity of autism/ASD, age 3-17 years? (ASDSevInd_21)	Would you describe this child's current autism or autism spectrum disorder as mild, moderate or severe, age 3-17 years?	Does not currently have autism or ASD; Current autism or ASD, rated mild; Current autism or ASD, rated moderate or severe
Someone living in the household smokes (Smoking_21)	Does anyone living in this child's household use cigarettes, cigars, or pipe tobacco?	Yes; No
Someone smokes inside the home (SmkInside_21)	Does anyone smoke inside this child's home?	No one smokes in the household; Someone smokes, not inside the house; Someone smokes inside the house
One or more health conditions (Cond2more24_21)	Does this child have current or lifelong health conditions from a list of 24 health conditions?<br>**This question was asked for:<br>epilepsy or seizure disorder; severe headaches including migraine (3-17 years), Tourette Syndrome (3-17 years), anxiety problems (3-17 years), depression (3-17 years), behavioral and conduct problem (3-17 years), developmental delay (3-17 years), intellectual disability (3-17 years), speech or other language disorder (3-17 years), learning disability (3-17 years), Autism or Autism Spectrum Disorder (ASD) (3-17	Does not have any current or lifelong health conditions; Has 1 current or lifelong health condition; Has 2 or more current or lifelong health conditions

	years), Attention Deficit Disorder or Attention-Deficit/Hyperactivity Disorder (ADD or ADHD) (3-17 years)	
Mental, emotional, developmental or behavioral problems, age 3-17 years (MEDB10ScrQ5_21)	Does this child have a mental, emotional, developmental or behavioral (MEDB) problem, age 3-17 years? **This question was asked for: Tourette Syndrome (3-17 years), anxiety problems (3-17 years), depression (3-17 years), behavioral and conduct problems (3-17 years), developmental delay (3-17 years), intellectual disability (3-17 years), speech or other language disorder (3-17 years), learning disability (also known as mental retardation) (3-17 years), Autism or Autism Spectrum Disorder (ASD) (3-17 years), Attention Deficit Disorder or Attention-Deficit/Hyperactivity Disorder (ADD or ADHD) (3-17 years)	Child has 1 or more reported MEDB problems and/or qualifies on CSHCN Screener emotional, behavioral or developmental criteria; Child does not currently have mental, emotional, developmental, or behavioral problems
Severity of current or lifelong conditions	How severe are this child's? conditions if the child has current or lifelong conditions?	Current condition, rated mild; Current condition, rated moderate/severe
